# Comparison of Perioperative Outcomes of Holmium Laser Enucleation of the Prostate for Standard (≤149 ml) Versus Very Large (≥150 ml) Prostate Glands: Retrospective Analysis of a Propensity Score Matched Cohort of 326 Patients

**DOI:** 10.1016/j.euros.2024.10.019

**Published:** 2024-11-18

**Authors:** Jacob Schmidt, Jorien Krediet, Holger Beutel, Ayoub Hidayat Allah, Nella Gagel, Isabel Lichy, Bernhard Ralla, Maha Ullmann, Robert Peters, Frank Friedersdorff, Martin Kanne

**Affiliations:** a Charité – Universitätsmedizin Berlin, corporate member of Freie Universität Berlin and Humboldt Universität zu Berlin, Department of Urology, Hindenburgdamm 30, 12203 Berlin, Germany; b Department of Urology, Evangelisches Krankenhaus Königin Elisabeth Herzberge, Berlin, Germany

**Keywords:** Prostatic hyperplasia, Holmium, Laser therapy, Prostate, Perioperative care, Complications, Propensity score matching

## Abstract

**Background and objective:**

Our objective was to evaluate whether a very large prostate volume significantly affects the incidence of perioperative complications and compromises outcomes among patients undergoing holmium laser enucleation of the prostate (HoLEP).

**Methods:**

We retrospectively analyzed data for 1815 adult patients who underwent HoLEP at Evangelisches Krankenhaus Königin Elisabeth Herzberge, Berlin, between January 2019 and May 2024. Patients were divided into two groups according to their prostate volume: ≤149 ml (group A) and ≥150 ml (group B). Propensity score matching on age, body mass index, American Society of Anesthesiologists physical status, and the presence of an indwelling catheter was used to balance baseline differences. A Mann-Whitney *U* test was used for comparison of continuous variables between the groups, and a χ^2^ test for comparison of categorical variables, with *p* < 0.05 considered statistically significant. Postoperative complications were assessed according to the Clavien-Dindo classification.

**Key findings and limitations:**

After propensity score matching, 163 matched cases per group were analyzed. Group B had significantly longer median total operative time (76 vs 47 min; *p* < 0.001), enucleation time (42 vs 26 min; *p* < 0.001), coagulation time (11 vs 6 min; *p* < 0.001), and morcellation time (15 vs 7 min; *p* < 0.001). Clavien-Dindo grade ≥IIIb complications (8.7% vs 1.2%; *p* = 0.02) and blood transfusion (2.5% vs 0%; *p* = 0.045) were significantly more frequent in group B. Catheterization time (1.9 vs 2.0 d; *p* = 0.01) and the proportion of patients with postoperative residual urine volume ≤50 ml (85.2% vs 80.2%; *p* = 0.18) were comparable between the groups. Limitations include the retrospective and single-center study design.

**Conclusions and clinical implications:**

Prostate volume ≥150 ml is associated with a longer operative time, a higher rate of major complications, and a more frequent need for blood transfusion. Therefore, HoLEP for prostate glands ≥150 ml should be performed in experienced high-volume centers.

**Patient summary:**

We compared outcomes of laser surgery for enlarged prostate glands of different sizes. We found that while the surgery is generally effective for very large prostates, it takes longer and has a higher risk of complications in comparison to more typical prostate sizes. However, this procedure is still the best treatment available for prostate enlargement and should be carried out in high-volume hospitals specializing in this treatment.

## Take Home Message

Holmium laser enucleation of the prostate (HoLEP) is effective for treating large prostates but is associated with longer operative times and a higher risk of complications for prostates ≥150 ml. Despite these challenges, HoLEP remains the preferred surgical treatment for benign prostatic enlargement and should be performed in experienced high-volume centers.

## 1 Introduction

Benign prostatic enlargement (BPE) is a prevalent condition affecting aging males [1], [2]. BPE often leads to lower urinary tract symptoms (LUTS), which have a significant impact on patients’ quality of life [3]. Various surgical techniques have been developed to treat BPE, among which holmium laser enucleation of the prostate (HoLEP) is increasingly used [4]. It has been shown that HoLEP is effective and safe across a wide range of prostate sizes, offering benefits such as shorter bladder irrigation time, catheter duration, and hospital stay, as well as lower complication rates and less blood loss in comparison to transurethral resection of the prostate (TURP) [1], [5], [6]. Although HoLEP is considered a size-independent procedure, there are limited studies on the outcome for glands ≥150 ml [6]. Extremely large prostates may present technical challenges, which can lead to longer operative times and potentially higher complication rates. Accordingly, the management of large prostates varies by institutions; in some centers, open simple prostatectomy (OSP) or robot-assisted simple prostatectomy (RASP) is still preferred over HoLEP [7], [8], [9]. Although Tay et al [7] did not find significant differences in the postoperative maximum flow rate, International Prostate Symptom Score (IPSS), or stricture and overall incontinence rates in a comparative study for men with prostate volume (PV) ≥150 ml versus <150 ml, their analysis of complications was sparse. Further results in the literature regarding complications after HoLEP for large prostates are heterogeneous. Tamalunas et al [10] did not found a higher complication rate for prostates >120 ml and concluded that HoLEP is a truly size-independent procedure. Tricard et al [8] reported an absence of severe bleeding events and a low rate of Clavien-Dindo grade III complications of 2.5% for their cohort with PV ≥150 ml. Other studies reported higher rates of bleeding events and blood transfusion for very large glands [2], [11], [12]. These discrepancies highlight the need for a detailed assessment of complications after HoLEP in patients with a very large prostate.

We performed a retrospective analysis of a propensity score matched (PSM) cohort to investigate perioperative outcomes of HoLEP in patients with PV ≤149 ml versus ≥150 ml. The primary objective was to evaluate whether PV ≥150 ml significantly affects the incidence of perioperative complications and compromises clinical outcomes.

## 2 Patients and methods

### 2.1 Patients

We retrospectively analyzed data for 1815 adult patients who underwent HoLEP at Evangelisches Krankenhaus Königin Elisabeth Herzberge (Berlin, Germany) between January 2019 and May 2024. The hospital is a certified center for endourological laser treatment (German ISA Din 9001), with >600 HoLEP cases performed annually. All patients were considered, including those with known prostate cancer in a palliative setting or previous surgical treatment such as TURP. Comprehensive patient data, including perioperative characteristics, sonographic findings, demographics, laboratory parameters, and clinical data, were collected from medical records. Perioperative complications were assessed according to the Clavien-Dindo classification [13]. The patients were divided into group A (PV ≤149 ml) and group B (PV ≥150 ml) according to preoperative PV determined via either abdominal or transrectal sonography. The enucleated tissue volume was determined on pathological examination.

### 2.2 Surgical technique and perioperative procedure

All patients were treated using a high-power HoPlus Laser System (generation 1 or 2) with an integrated morcellator (JenaSurgical, Jena, Germany; settings: 3 J and 40 Hz = 120 W; or 1.5 J and 40 Hz = 60 W, respectively) by two experienced surgeons (>500 cases and >1500 cases). En-bloc enucleation was the most common surgical technique, and an early apical release incision was regularly used to avoid shear forces on the sphincter fibers as early as possible [14]. Other HoLEP techniques used in our cohort included two-lobe and three-lobe enucleation. A urine sample for microbial testing was collected from each patient before surgery. If the results were positive and the patient was asymptomatic, oral antibiotic treatment was started from the morning of the day of surgery for at least 5 d. In cases with resistant bacteria, we started intravenous treatment a few days before surgery. For patients with a symptomatic urinary tract infection, we suspended surgery. If the urine culture was negative, we administered a single dose of ceftriaxone perioperatively.

All anticoagulant medications, except for acetylsalicylic acid 100 mg/d, were discontinued perioperatively. Bridging was performed according to the recommendations of the attending physician who prescribed the medication.

### 2.3 Statistical analysis

**Fig. 1:**
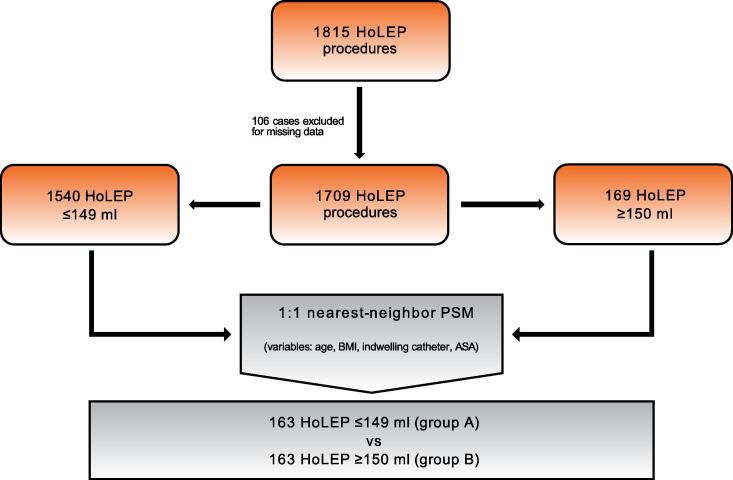
Flowchart of the selection process for patients for propensity score matching (PSM) by age, body mass index (BMI), indwelling catheter use, and American Society of Anesthesiologists (ASA) score.

We used PSM to balance baseline differences between the two groups. After excluding 106 cases because of missing data, 1709 cases remained. We performed 1:1 PSM with a caliper width of 0.01, with matching by age, body mass index, American Society of Anesthesiologists (ASA) physical status classification, and presence of an indwelling catheter, which resulted in 326 matched cases (Fig. 1). Statistical analysis was performed using Microsoft Office Excel 2022 (Microsoft Corporation, Redmond, WA, USA) and IBM SPSS v29 software (IBM Corporation, Armonk, NY, USA). Mann-Whitney *U* tests were used for between-group comparison of continuous variables and a χ^2^ test for comparison of categorical variables, with *p* < 0.05 considered statistically significant.

## 3 Results

Characteristics of the overall cohort before PSM are shown in Supplementary Table 1. After PSM, demographic characteristics were comparable between the groups (Table 1). The median age was 73 (range 52–91) yr in group A and 74 (range 54–94) yr in group B (*p* = 0.81). Median body mass index was 27.2 (range 17.4–39.9) kg/m^2^ in group A and 26.9 (range 20.5–39.8) kg/m^2^ in group B (*p* = 0.34). The distribution of ASA scores for groups A versus group B was as follows: score 1, 14 patients (8.6%) versus 18 patients (8%); score 2, 55 patients (37.7%) versus 63 patients (38%); and score 3, 94 patients (57.7%) versus 88 patients (54%), with no significant difference B (*p* = 0.72). An indwelling catheter was present in 89 patients (54.6%) in group A and 92 patients (56.4%) in group B (*p* = 0.74).

**Table 1:** Demographic and clinical characteristics of the matched study groups

Parameter	PV ≤149 ml (*n* = 163)	PV ≥150 ml (*n* = 163)	*p* value a
Median age, yr (range)	73 (52–91)	74 (54–94)	0.81
Median body mass index, kg/m^2^ (range)	27.2 (17.4-39.9)	26.9 (20.5–39.8)	0.34
ASA score, *n* (%)			0.72
ASA 1	14 (8.6)	13 (8%)	
ASA 2	55 (33.7)	62 (38%)	
ASA 3	94 (57.7)	88 (54%)	
Indwelling catheter, *n* (%)	89 (54.6)	92 (56.4%)	0.74

PV = prostate volume; ASA = American Society of Anesthesiologists.

a Mann-Whitney *U* test or χ^2^ test.

As shown in Table 2, there was a significant difference in PV on sonography, with a median of 61 (range 15–140) ml in group A and 170 (range 150–400) ml in group B (*p* < 0.001). Group A included seven patients (4.3%) with a normal-sized prostate of <30 ml. Transrectal sonography was performed in 32 patients (19.8%) in group A and 23 patients (14.1%) in group B (*p* = 0.18); for the remaining patients, PV was determined via abdominal sonography. Median IPSS was significantly higher in group A (21, range 2–35) than in group B (18, range 1–33; *p* = 0.005). The preoperative residual urine volume (RUV) distribution was similar between the groups (Table 2).

**Table 2:** Perioperative characteristics of the matched study groups

Parameter	PV ≤149 ml (*n* = 163)	PV ≥150 ml (*n* = 163)	*p* value a
**Preoperative characteristics**			
Median PV on ultrasound, ml (range)	61 (15–140)	170 (150–400)	**<0.001**
Median International Prostate Symptom Score (range)	21 (2–35)	18 (1–33)	**0.005**
Median prostate-specific antigen, ng/ml (range)	3.4 (0–162)	8.0 (1–152)	**<0.001**
History of urinary tract infection, *n* (%)	95 (58.3)	99 (61.5)	0.56
Preoperative residual urine status, *n* (%)			0.27
Chronic urinary retention/indwelling catheter	86 (53.8)	88 (54.7)	
≤50 ml	29 (18.1)	17 (10.6)	
51–100 ml	10 (6.3)	17 (10.6)	
101–200 ml	27 (16.9)	30 (18.6)	
>200 ml	8 (5)	9 (5.6)	
Anticoagulant/platelet medication, *n* (%)			
Acetylsalicylic acid monotherapy	46 (28.2)	30 (18.4)	**0.04**
Dual antiplatelet therapy	2 (1.2)	1 (0.6)	0.56
New oral anticoagulant/LMWH	35 (21.5)	23 (14.1)	0.08
Phenprocoumon	5 (3.1)	6 (3.7)	0.76
Anti-obstructive medication, *n* (%)			
α-Blocker	81 (69.2)	68 (58.6)	0.09
5α-Reductase inhibitor	3 (2.6)	4 (3.4)	0.63
α-Blocker + 5α-reductase inhibitor	23 (19.7)	38 (33.5)	**0.02**
Other	10 (8.5)	8 (6.9)	0.63
**Intraoperative characteristics**			
Median operative time, min (range)	47 (15–119)	76 (30–175)	**<0.001**
Median enucleation time, min (range)	26 (8–58)	42 (15–130)	**<0.001**
Median coagulation time, min (range)	6 (0–22)	11 (0–60)	**<0.001**
Median morcellation time, min (range)	7 (1–25)	15 (2–80)	**<0.001**
Median enucleated tissue volume, ml (range)	50 (2–134)	131 (27–460)	**<0.001**
**Postoperative parameters**			
Median catheterization duration, d (range)	1.9 (0.9–7.6)	2 (1.6–9)	0.10
Postoperative residual urine status, *n* (%)			0.18
Need for catheterization	0	1 (0.6)	
≤50 ml	130 (80.2)	138 (85.2)	
51–100 ml	21 (13)	20 (12.3)	
101–200 ml	9 (5.6)	3 (1.9)	
>200 ml	2 (1.2)	0	

PV = prostate volume; LMWH = low-molecular-weight heparin.

a Mann-Whitney *U* test or χ^2^ test. Significant p values are denoted in bold font.

Acetylsalicylic acid was used by 46 patients (28.2%) in group A and 30 patients (18.4%) in group B (*p* = 0.04). Use of new oral anticoagulants (NOACs), low-molecular-weight heparin (LMWH), and phenprocoumon use similar between the groups (Table 2). Antiobstructive medications included α-blockers (ABs), 5α-reductase inhibitors (5-ARI), and a combination of the two classes (AB + 5-ARI, 19.7% vs 33.5%; *p* = 0.02). Other drugs included herbal medications, phosphodiesterase type 5 inhibitors, and anticholinergic drugs.

The median operative time was significantly shorter for group A at 47 (range 15–119) min, in comparison to 76 (range 30–175) min for group B (*p* < 0.001). The median enucleation time (26 vs 42 min), coagulation time (6 vs 11 min), and morcellation time (7 vs 15 min) were also significantly shorter for group A (all *p* < 0.001). The median enucleated tissue volume was higher in group B (131 ml, range 27–460) than in group A (50 ml, range 2–134 ml; *p* < 0.001).

After surgery, the catheterization duration was 2 (range 1.6–9) d in group B and 1.9 (range 0.9–7.6) d in group A (*p* = 0.10). Postoperative RUV ≤50 ml was achieved in 130 patients (80.2%) in group A and 138 (85.2%) in group B, RUV 51–100 ml in 21 patients (13%) in group A and 20 (12.3%) in group B, RUV 101–200 ml in nine patients (5.6%) in group A and three (1.9%) in group B, and RUV >200 ml in two patients (1.2%) in group A and none in group B (*p* = 0.18).

As shown in Table 3, complications were observed in 24 patients (14.9%) in group A versus 35 patients (21.6%) in group B (*p* = 0.12). The number of complications per patient was significantly higher in group B, with 11 patients (7.1%) experiencing multiple complications, compared to no case with multiple complications in group A (*p* = 0.01). Major complications (grades IIIb and IVa) were more frequent in group B (*p* = 0.02). Grade II complications occurred in 22 patients (13.7%) in group A and 21 patients (13.0%) in group B. Grade IIIb complications requiring surgical intervention under general anesthesia were observed in two patients (1.2%) in group A and ten patients (6.2%) in group B [13]. Grade IVa complications requiring intensive care management occurred in four patients (2.5%) in group B and none in group A. Specific complications included fever in ten patients (6.2%) in group A and 15 (9.3%) in group B (*p* = 0.31), recatheterization in 12 patients (7.5%) in group A and five (3.1%) in group B (*p* = 0.08), and macrohematuria with prolonged catheterization in three patients (1.9%) in group B and none in group A (*p* = 0.88). Blood transfusion was required in four patients (2.5%) in group B and none in group A (*p* = 0.045). One patient in group B experienced intraoperative prostatic capsule perforation with free pelvic fluid, which was conservatively treated with prolonged catheterization. Surgical revision was needed significantly more frequently for PV ≥150 ml, with 11 patients (6.8%) in group B in comparison to two (1.2%) in group A (*p* = 0.01). In group A, two revisions were necessary for postoperative bleeding. In group B, there were eight cases of postoperative bleeding, one second-look morcellation for a lost fragment, one incomplete HoLEP because of technical issues, and one aborted procedure because of septic embolization necessitating reoperation. Intensive care was not required in group A, but was needed for four cases (2.5%) in group B (*p* = 0.045). One patient experienced septic embolization during HoLEP, leading to an aborted procedure, transfer to the intensive care unit (ICU), and a second surgery. Another patient had intraoperative upper abdominal pain and nausea requiring visceral surgery and ICU admission. One patient with coronary artery disease and one with hyponatremia required a 1-d ICU stay for monitoring. Incidental prostate cancer was found in 37 patients (22.7%) in group A and in 20 patients (12.3%) in group B (*p* = 0.01). Comparison for the different HoLEP techniques used revealed no significant differences in the distribution of complications, transfusions, and intensive medical care, as shown in Supplementary Table 1.

**Table 3:** Incidence of complications in the matched study groups

Parameter	PV ≤149 ml (*n* = 163)	PV ≥150 ml (*n* = 163)	*p* value a
Patients with complications, *n* (%)	24 (14.9)	35 (21.6)	0.12
Number of complications, *n* (%)			**0.01**
One complication	24 (14.9)	24 (14.9.)	
Two complications	0	10 (6.5)	
Three complications	0	1 (0.6)	
Highest Clavien-Dindo grade, *n* (%)			**0.02**
Grade II	22 (13.7)	21 (13)	
Grade IIIb	2 (1.2)	10 (6.2)	
Grade IVa	0	4 (2.5)	
Specific complications, *n* (%)			
Fever	10 (6.2)	15 (9.3)	0.31
Recatheterization	12 (7.5)	5 (3.1)	0.08
Macrohematuria with prolonged catheterization	0	3 (1.9)	0.88
Blood transfusion	0	4 (2.5)	**0.045**
Prostatic capsule perforation with free abdominal fluid	0	1 (0.6)	0.32
Revision	2 (1.2)	11 (6.8)	**0.01**
Intensive medical care	0	4 (2.5)	**0.045**
Incidental prostate cancer	37 (22.7)	20 (12.3)	**0.01**

PV = prostate volume.

a χ^2^ test. Significant *p* values are denoted in bold font.

## 4 Discussion

It has been established that HoLEP is an effective surgical technique for BPE treatment, offering advantages such as minimal invasiveness, lower blood loss, and shorter hospital stays in comparison to traditional TURP or OSP [4], [15]. Although HoLEP is considered size-independent, the influence of very large gland size on HoLEP outcomes remains a subject of clinical interest. The aim of our retrospective analysis was to compare perioperative outcomes for PV of ≤149 ml versus ≥150 ml.

Demographic characteristics were balanced between the study groups after PSM, ensuring that any differences in outcomes could be attributed to prostate size alone. Interestingly, preoperative IPSS was significantly higher in group A, suggesting that patients with a smaller prostate experienced more severe LUTS. This may be explained by the findings of Kim et al [16], who found a significant association between PV and LUTS only for certain components, such as central volume and nocturia, and not necessarily for total gland size. We treated seven patients with PV <30 ml; despite a small gland size, these patients are considered for HoLEP at our institution if either drug therapy for a proven localized obstruction has been unsuccessful or an obstruction is still present after a previous surgical prostate reduction. Another explanation could be that significantly more patients in group B were taking an AB + 5-ARI combination, which could reduce the severity of LUTS [17]. Acetylsalicylic acid use was prevalent among the study population (28.2% of group A and 18.4% of group B; *p* = 0.04). In addition, 21.5% of the patients in group A and 14.1% in group B were on an NOAC or LMWH (*p* = 0.08). Interestingly, the higher prevalence of antiplatelet/anticoagulant use in group A did not translate into a higher rate of bleeding complications. This confirms that HoLEP is a safe procedure with a generally low risk of bleeding and, unlike TURP, can be performed in patients on anticoagulants [5], [18].

The efficacy of HoLEP in reducing RUV was evident in our study. Postoperative RUV ≤50 ml was achieved in the majority of patients in both groups, indicating effective bladder emptying and comprehensive removal of obstructive tissue. The comparable postoperative RUV results with equivalent catheterization time support the perception that HoLEP is a size-independent procedure [7], [10].

As expected, there were significant differences in operative times (total operative time, and enucleation, coagulation, and morcellation times) between the two groups, with longer times in group B. These differences are in accordance with literature results and can probably be attributed to the greater technical complexity associated with very large glands, which require extensive enucleation and morcellation [7]. Our analysis also revealed significant differences in the incidence of perioperative complications. The group with PV ≥150 ml had higher rates of major complications (grades IIIb and IVa) and blood transfusion, and these patients were more likely to experience multiple complications. Revisions were more common in group B, primarily for postoperative bleeding. However, it is important to note that these revisions are usually performed on the same day and the length of hospitalization or catheterization is not necessarily extended. It is also important to consider that our cohort had a substantial proportion of patients with ASA ≥2 and was thus probably more susceptible to complications than cohorts in comparable studies [9], [19]. This is also reflected in the fact that more than 50% of our patients had an indwelling catheter at the time of surgery, which is an unusually high rate for a BPE cohort. Our results are concordant with the findings of Porto et al [6], who identified size as a risk factor for bleeding events and blood transfusion in their literature review, but differ from studies such as those by Tamalunas et al [10] and Tricard et al [8]. Other specific postoperative complications, including fever, recatheterization, and macrohematuria with prolonged catheterization, were also more frequent in group B, although the differences in comparison to group A were not statistically significant. Therefore, our results suggest that HoLEP is a functionally size-independent procedure, but particular attention should be paid to the perioperative risk of complications for PV ≥150 ml in a more vulnerable cohort such as our study population. This raises the question of whether other treatment options may be more appropriate for very large glands. In this context, Lee et al [20] found that for glands of 150 ml, HoLEP was associated with the shortest hospitalization and catheterization times in comparison to RASP and OSP. The catheterization times in their study were 9.9 d after OSP and 11.2 d after RASP, which are substantially longer than the ∼2 d after HoLEP in our study. Although hospital length of stay was not recorded for our cohort, it is standard practice to discharge patients on the day after catheter removal, confirming that HoLEP is an effective and cost-effective procedure in comparison to OSP or RASP [21]. Lee et al [20] reported a transfusion rate of 47% after OSP, which appears to be substantially higher than the rate for our HoLEP cohort (2.5% in group B). Naspro et al [22] observed a significantly higher transfusion rate after OSP for 120-ml glands than for HoLEP for 110-ml glands with operative times equivalent those for group B in our study. Jones et al [23] did not find a significant difference in the complication rate between HoLEP and OSP in their meta analysis for a median gland size of 120 ml. Grosso et al [19] reported a lower rate of early complications and a shorter catheterization time for HoLEP in comparison to RASP. For their HoLEP group, the transfusion rate was 4.2% and the catheterization time was 4 d, which are both greater than the corresponding outcomes in our study. It should be emphasized that the median gland size of 170 ml in group B is substantially larger than in the majority of the aforementioned studies, and a substantial number of patients had severe comorbidity, with ASA scores of 2–3. Therefore, although surgical treatment options should be carefully weighed given the higher risk of complications for glands ≥150 ml, and a cautious approach is needed for preoperative patient assessment, we suggest that HoLEP is the best treatment option for very large glands because of the shorter operative time in comparison to OSP and RASP and the favorable perioperative outcomes.

Our analysis has several limitations. The retrospective and single-center nature of the study may introduce inherent biases related to surgical expertise and patient selection. Moreover, although we used PSM to balance baseline characteristics, other confounders may have influenced the outcomes, such as differences in IPSS, as discussed above. In addition, no strict inclusion or exclusion criteria for patients were applied, so patients with previous TURP or prostate cancer were also included, which may bias the results. Another possible confounder is the use of different HoLEP techniques. Lastly, the lack of long-term follow-up limits the informative value of functional outcomes.

## 5 Conclusions

Prostate size ≥150 ml can influence perioperative complication and blood transfusion rates. Therefore, HoLEP for very large prostate glands should be performed in experienced high-volume centers and a prudent approach should be used for patient assessment. Our findings indicate that effective bladder emptying can be achieved following HoLEP for glands ≥150 ml, as evidenced by low RUV. Although the study results cannot be directly extrapolated to other surgical techniques, HoLEP for glands ≥150 ml remains an excellent treatment option with the many advantages of a minimally invasive procedure in comparison to OSP and RASP for prostate enlargement.

  ***Author contributions***: Jacob Schmidt had full access to all the data in the study and takes responsibility for the integrity of the data and the accuracy of the data analysis.

  *Study concept and design*: Schmidt, Kanne, Friedersdorff.

*Acquisition of data*: Kanne, Krediet, Beutel, Allah, Gagel.

*Analysis and interpretation of data*: Schmidt, Kanne.

*Drafting of the manuscript*: Schmidt.

*Critical revision of the manuscript for important intellectual content*: Schmidt, Kanne, Friedersdorff, Lichy, Ralla, Ullmann, Peters.

*Statistical analysis*: Schmidt.

*Obtaining funding*: None.

*Administrative, technical, or material support*: Friedersdorff, Kanne.

*Supervision*: Kanne, Friedersdorff.

*Other*: None.

  ***Financial disclosures:*** Jacob Schmidt certifies that all conflicts of interest, including specific financial interests and relationships and affiliations relevant to the subject matter or materials discussed in the manuscript (eg, employment/affiliation, grants or funding, consultancies, honoraria, stock ownership or options, expert testimony, royalties, or patents filed, received, or pending), are the following: None.

  ***Funding/Support and role of the sponsor*:** None.

  ***Ethics considerations***: This study is exempt from the local ethics committee approval because of the retrospective, noninterventional design and because patient data confidentiality and privacy were maintained at all times. The study was performed in accordance with the principles of the Declaration of Helsinki.

  ***Data sharing statement:*** The data that support the findings of this study are not openly available for reasons of sensitivity but are available from the corresponding author on reasonable request.
