# Supplementary material for: Comparison of Perioperative Outcomes of Holmium Laser Enucleation of the Prostate for Standard (≤149 ml) Versus Very Large (≥150 ml) Prostate Glands: Retrospective Analysis of a Propensity Score Matched Cohort of 326 Patients
Source: Eur Urol Open Sci. 2024 Nov 18;71:15–21. doi: 10.1016/j.euros.2024.10.019 (PMC11617294; doi:10.1016/j.euros.2024.10.019)
Supplement: Supplementary Data 1 [file mmc1.docx]

**Supplementary Table 1.** Comparison of different HoLEP techniques; values are shown as median (range) or number (percentage of the group); *p-value <0.05 in Mann-Whitney U- or Chi-square test.

| **Characteristics** | **group A (n=163)**  ≤ 149 ml | **group B (n=163)**  ≥ 150 ml | **P** |
| --- | --- | --- | --- |
| **HoLEP technique** |  |  |  |
| Unknown | 13 (8%) | 10 (6.1%) |  |
| En-bloc enucleation | 96 (58.9%) | 70 (42.9%) |  |
| Two-/three-lobe enucleation | 54 (33.1%) | 83 (50.9%) |  |
| **Operative time^†^ (min)** |  |  |  |
| En-bloc enucleation | 42 (15-119) | 65 (30-170) | *<0.001 |
| Two-/three-lobe enucleation | 51 (23-102) | 83 (37-175) | *<0.001 |
| **En-bloc enucleation^†^** |  |  |  |
| Clavien-Dindo |  |  | 0.30 |
| II | 7 (7.4%) | 9 (13%) |  |
| IIIb | 2 (2.1%) | 3 (4.3%) |  |
| IVa | 0 | 1 (1.4%) |  |
| Blood transfusion | 0 | 1 (1.4%) | 0.24 |
| Revision | 2 (2.1%) | 3 (4.3%) | 0.42 |
| Intensive medical care | 0 | 1 (1.4%) | 0.24 |
| **Two-/three-lobe^†^** |  |  |  |
| Clavien-Dindo |  |  | 0.17 |
| II | 19 (12.8%) | 21 (13.8) |  |
| IIIb | 2 (1.4%) | 7 (4.6%) |  |
| IVa | 0 | 3 (2.0%) |  |
| Blood transfusion | 0 | 2 (2.4%) | 0.25 |
| Revision | 0 | 4 (4.8%) | 0.10 |
| Intensive medical care | 0 | 2 (2.4%) | 0.25 |

^†^Cases with unknown HoLEP technique were excluded

**Supplementary Table 2.** Characteristics of the whole cohort before propensity score matching; values are shown as median (range) or number (percentage of the group); *p-value <0.05 in Mann-Whitney U- or Chi-square test.

|  | **group A (n=1646)**  ≤ 149 ml | **group B (n=169)**  ≥ 150 ml | **P** |
| --- | --- | --- | --- |
| **Preoperative characteristics** |  |  |  |
| Age – median (years) (range) | 72 (44-94) | 75 (54-94) | 0.10 |
| BMI (kg/m^2^) (range) | 26.6 (14.43-48.1) | 26.9 (20.5-39.8) | 0.09 |
| PSA (ng/ml) (range) | 3.4 (0-289) | 7.9 (1-152) | ***<0.001** |
| IPSS (range) | 22 (2-35) | 18 (1-33) | ***0.009** |
| Sonographically determined prostate volume (ml) (range) | 65 (15-146) | 170 (150-400) | ***<0.001** |
| ASA-score (n) | 1575 | 164 | 0.831 |
| ASA 1 | 92 (5.8%) | 13 (7.9%) |  |
| ASA 2 | 580 (36.8%) | 62 (37.8%) |  |
| ASA 3 | 902 (57.7%) | 89 (54.3%) |  |
| ASA 4 | 1 (0.1%) | 0 |  |
| Indwelling catheter | 676/1625 (41.6%) | 97/169 (57.4%) | ***<0.001** |
| History of urinary tract infection | 821/1613 (50.9%) | 104/167 (62.3%) | ***<0.001** |
| **Intraoperative characteristics** |  |  |  |
| Operative time (min) (range) | 44 (4-190) | 77 (30-175) | ***<0.001** |
| Enucleation time (min) (range) | 25 (2-90) | 42 (15-130) | ***<0.001** |
| Coagulation time (min) (range) | 6 (0-50) | 11 (0-60) | ***<0.001** |
| Morcellation time (min) (range) | 7 (0-60) | 15 (2-80) | ***<0.001** |
| Enucleated tissue volume (ml) (range) | 45 (1-270) | 131 (27-460) | ***<0.001** |
| **Postoperative parameters** |  |  |  |
| Duration of catheterization (d) (range) | 1.96 (0.75-41.6) | 2 (1.6-41.6) | 0.94 |
| Residual urine (postoperative) | 1619 | 167 | 0.11 |
| Need for catheterization | 16 (1%) | 1 (0.6%) |  |
| ≤50 ml | 1340 (82.8%) | 141 (84.4%) |  |
| 51-100 ml | 191 (11.8%) | 21 (12.4%) |  |
| 101-200 ml | 61 (3.8%) | 4 (2.4%) |  |
| >200 ml | 11 (0.7%) | 0 |  |
| Patients with complications | 247 (15.2%) | 39 (23.2%) | ***0.07** |
| Clavien-Dindo Grade (highest) | 1628 | 169 | ***<0.001** |
| II | 200 (12.3%) | 24 (14.2%) |  |
| IIIb | 41 (2.5%) | 11 (6.5%) |  |
| IVa | 5 (0.3%) | 4 (2.4%) |  |
| V | 1 (0.1%) | 0 |  |

**Abbrevations:** ASA American Society of Anesthesia, BMI Body Mass Index
